# Safety and Efficacy of Ozanimod in Patients With Moderately to Severely Active Ulcerative Colitis Stratified by Age

**DOI:** 10.1093/ibd/izaf258

**Published:** 2026-03-01

**Authors:** Adam S. Faye, David T. Rubin, Corey A. Siegel, Millie D. Long, Nabeel Khan, Silvio Danese, Peter M. Irving, Raymond K. Cross, Irina Blumenstein, Alessandro Armuzzi, Sara N. Horst, Axel Dignass, Taku Kobayashi, Garrett Lawlor, Anthony Krakovich, AnnKatrin Petersen, Zhaohui Liu, Dong Wang, Anjali Jain, Ashwin N. Ananthakrishnan, João Sabino

**Affiliations:** 1 NYU Langone Health, New York, NY, United States; 2 University of Chicago Medicine Inflammatory Bowel Disease Center, Chicago, IL, United States; 3 Walter and Carole Young Center for Digestive Health, Dartmouth-Hitchcock Medical Center, Lebanon, NH, United States; 4 Department of Medicine, University of North Carolina at Chapel Hill, Chapel Hill, NC, United States; 5 Corporal Michael J. Crescenz VA Medical Center, Philadelphia, PA, United States; 6 Department of Gastroenterology and Endoscopy, IRCCS Ospedale San Raffaele and University Vita-Salute San Raffaele, Milan, Italy; 7 Guy’s and St Thomas’ NHS Foundation Trust, London, United Kingdom; 8 Institute for Digestive Health and Liver Disease, Mercy Medical Center, Baltimore, MD, United States; 9 Medical Clinic 1, Frankfurt University Hospital, Goethe University Frankfurt, Frankfurt, Germany; 10 IBD Center, IRCCS Humanitas Research Hospital, Rozzano, Italy; 11 Department of Biomedical Sciences, Humanitas University, Pieve Emanuele, Italy; 12 Vanderbilt University Medical Center, Nashville, TN, United States; 13 Agaplesion Markus Hospital, Goethe University, Frankfurt, Germany; 14 Kitasato University Kitasato Institute Hospital, Tokyo, Japan; 15 Bristol Myers Squibb, Princeton, NJ, United States; 16 Massachusetts General Hospital, Boston, MA, United States; 17 Department of Gastroenterology, University Hospitals Leuven, Leuven, Belgium

**Keywords:** inflammatory bowel disease, ulcerative colitis

## Abstract

**Background:**

Older adults with ulcerative colitis (UC) have unique treatment challenges. Ozanimod is approved for the treatment of moderately to severely active UC in adults based on the phase 3 True North (TN) study results. Here, we analyzed the impact of patient age on ozanimod safety and efficacy in TN and during the open-label extension (OLE).

**Methods:**

Patients were stratified by age at TN baseline: <40, 40 to 60, and >60 years (cutoff: 75 years). Safety was evaluated in all patients during TN and the OLE; efficacy was assessed at weeks 10 and 52 in TN and up to OLE week 190 in patients who entered as TN week 52 ozanimod clinical responders.

**Results:**

Of 1012 patients analyzed, 492 were <40 years of age, 404 were 40 to 60 years of age, and 116 were >60 years of age. Infection, malignancy, cardiac events, and macular edema were low throughout TN across all ages. Exposure-adjusted incidence rates (EAIRs) of opportunistic and serious infections increased with age during the OLE. Patients ≥40 years of age had higher hypertension EAIRs than those <40 years of age, but EAIRs of other cardiovascular TEAEs were low. No cases of progressive multifocal leukoencephalopathy occurred over 242 weeks of ozanimod exposure. Efficacy rates for evaluated clinical and mucosal endpoints at weeks 10 and 52 with ozanimod were generally consistent across age groups with the overall population; similar trends were observed in the OLE.

**Conclusions:**

Ozanimod safety was similar and efficacy was generally comparable across age groups, although statistical significance vs placebo was not achieved in patients >60 years of age.

## Lay Summary

Immunosuppressive treatment can be associated with safety concerns in older patients with ulcerative colitis. We found that ozanimod safety and efficacy were generally consistent across age groups, supporting ozanimod as an oral treatment option for older adults with ulcerative colitis.

## Introduction

Ulcerative colitis (UC) is a type of inflammatory bowel disease (IBD) with increasing worldwide prevalence.^1,2^ Although the incidence of UC, including older-onset UC, has stabilized in recent years in Western countries, the prevalence of IBD in older adults (≥60–65 years of age) is continuing to rise as an increasing number of adults with earlier-onset disease are aging.^3–7^

There are unique challenges associated with treating older patients with IBD with regard to safety. Treatment of older patients with IBD can involve an overreliance on corticosteroids, which carry risks, such as cardiometabolic, ophthalmic, and cognitive conditions; infections; and osteoporosis in older adults.^5,8,9^ There is also hesitancy in using IBD therapies, such as tumor necrosis factor (TNF) inhibitors, Janus kinase inhibitors, and thiopurines, as these have been shown to increase the risk of infection and malignancy in older patients.^10–15^ Additionally, older patients with IBD have been found to have greater risk of adverse events (AEs) (eg, infection, major adverse cardiovascular events, thromboembolic complications, malignancies), as they have higher rates of comorbidities, particularly cardiovascular disease, and polypharmacy.^5,6,16–23^ This increased susceptibility to AEs can make physicians reluctant to prescribe advanced therapies for older patients.^6,24^ As a result, older patients are less likely to receive advanced therapies.^19^ However, evidence suggests that inadequately treated disease may lead to worse clinical outcomes as compared with the potential adverse effects from treatment with advanced therapies.^24^

Therefore, it is important to treat older adults with moderate to severe disease with advanced therapies and to identify advanced therapies that are convenient, efficacious, and safe in this population. Ozanimod is an oral small molecule and selective sphingosine 1-phosphate (S1P) receptor 1 and 5 modulator approved for the treatment of patients with moderately to severely active UC.^25,26^ Ozanimod was found to be efficacious and well tolerated for up to 52 weeks in patients with moderately to severely active UC in the phase 3 True North study.^27^ Interim analyses of the True North open-label extension (OLE) have demonstrated sustained efficacy over ∼5 years of continuous ozanimod treatment.^28^ Ozanimod has not yet been evaluated specifically in older populations. However, the potential for cardiac and hepatic adverse reactions to ozanimod due to reduced cardiac and hepatic function in older patients makes understanding ozanimod safety particularly important in this population.^25^ Thus, the objective of this analysis was to evaluate the impact of patient age on ozanimod safety and efficacy in True North and the OLE.

## Methods

### Study design

The study designs of True North (NCT02435992) and the True North OLE (NCT02531126) have been previously described (Figure S1).^27,29^ Briefly, True North was a 52-week, randomized, double-blind, placebo-controlled phase 3 trial. In a 10-week induction period, patients in cohort 1 were randomized 2:1 to receive ozanimod 0.92 mg or placebo. In cohort 2, patients received open-label ozanimod. Ozanimod-treated patients who achieved clinical response at week 10 were eligible to enter a 42-week maintenance period in which patients were rerandomized 1:1 to receive ozanimod or placebo in a double-blind manner. Patients who did not achieve clinical response at week 10, experienced disease relapse (ie, an increase in partial Mayo score ≥2 points compared with week 10 and an absolute partial Mayo score ≥4 points and a Mayo endoscopy subscore [MES] of ≥2 points) in the maintenance period, or completed week 52 were eligible to enter the OLE, in which all patients received ozanimod 0.92 mg.

### Patients

Detailed inclusion and exclusion criteria for True North were also previously described.^27^ Patients eligible to enroll in True North were 18 to 75 years of age; this analysis grouped patients by age at True North baseline: <40 years, 40 to 60 years, and >60 years. Cutoffs were selected based on clinically relevant age categories in the literature.^30–32^ Patients had moderately to severely active UC, defined as a total Mayo score of 6 to 12, with an MES of ≥2, a rectal bleeding subscore (RBS) of ≥1, and a stool frequency subscore (SFS) of ≥1. Patients were excluded for clinically relevant cardiovascular conditions or a history of diabetes, uveitis, macular edema, or cancer. Clinically relevant cardiovascular conditions included history or presence of the following: (1) recent (within the last 6 months) occurrence of myocardial infarction, unstable angina, stroke, transient ischemic attack, decompensated heart failure requiring hospitalization, class III/IV heart failure, sick sinus syndrome, or severe untreated sleep apnea; (2) prolonged Fridericia’s corrected QT interval (QTcF) (QTcF >450 ms for males and QTcF >470 ms for females) or at additional risk for QT interval prolongation (eg, hypokalemia, hypomagnesemia, congenital long QT syndrome); or (3) resting heart rate <55 beats/min when taking vital signs as part of a physical exam at screening.

### Outcomes

#### Safety assessments

Safety assessments included treatment-emergent adverse events (TEAEs) during the True North induction period and maintenance period and the OLE in all patients who entered the OLE. In addition, herpes zoster cases and vaccination status of patients with herpes zoster, absolute lymphocyte count (ALC) reductions and hepatic enzyme elevations during the True North induction and maintenance periods and the OLE, and the relationship of ALC reductions to treatment discontinuation and infections were assessed.

#### Laboratory assessments

Laboratory assessments included mean ALC in the True North induction and maintenance periods and mean ALC in the OLE through OLE week 190 in patients who received ozanimod during the True North induction and maintenance periods, completed maintenance with clinical response at week 52, and subsequently entered the OLE.

#### Efficacy

Endoscopic and histologic endpoints were determined by a central reader who used blinded videos of endoscopic procedures and preserved biopsy samples, respectively. Rectal bleeding and stool frequency were reported by patients in an electronic diary. The primary efficacy endpoint was the percentage of patients with clinical remission, assessed by the 3-component modified Mayo score at weeks 10 and 52. Clinical remission was defined as follows: an RBS of 0; an SFS of ≤1, with a decrease of ≥1 point from baseline; and an MES of ≤1 (all on scales from 0 [none] to 3 [most severe]). Key secondary endpoints included clinical response (ie, reduction from baseline in the modified Mayo score of ≥2 points and ≥35%, and a reduction from baseline in RBS of ≥1 point or an absolute RBS of ≤1 point), endoscopic improvement (MES of ≤1 point without friability), and mucosal healing (MES of ≤1 point without friability and a Geboes score <2.0), evaluated at weeks 10 and 52, as well as corticosteroid-free remission (ie, remission with no corticosteroid use for ≥12 weeks) at week 52. Other prespecified endpoints included histologic remission (Geboes score <2.0). All endpoints were also evaluated throughout the OLE in patients who received ozanimod during the True North induction and maintenance periods, completed maintenance with clinical response at week 52, and subsequently entered the OLE.

### Statistical analysis

This is a post hoc analysis of True North and the OLE; the data cutoff from the OLE (June 30, 2023) comes from an interim analysis, as the analysis was initiated before database lock. Efficacy was assessed in all patients who received ≥1 doses of study drug. In the True North induction period, statistical comparisons of efficacy endpoints were performed in cohort 1 and descriptive statistics were performed in cohort 2; in the maintenance period, statistical comparisons of efficacy endpoints were performed to compare the ozanimod treatment group with the group that switched to placebo during maintenance. Adjusted treatment differences and nominal *P* values for comparison between the ozanimod and placebo groups were calculated using the Cochran-Mantel-Haenszel test, and were stratified by corticosteroid use at screening and prior anti-TNF use (week 10 comparisons) or by week 10 remission status and week 10 corticosteroid use (week 52 comparisons). Missing data in the induction and maintenance periods were handled using nonresponder imputation (NRI). OLE data were summarized descriptively using observed case and NRI analyses.

### Ethics

This analysis adhered to the Good Clinical Practice guidelines and the ethical principles outlined in the Declaration of Helsinki. Investigators at each study site obtained protocol and informed consent approval by an institutional review board or independent ethics committee. Written consent was obtained from each patient in the study, which was sponsored by Bristol Myers Squibb.

## Results

### Baseline demographic and disease characteristics

A total of 1012 patients were included, of whom 492 were <40 years of age, 404 were 40 to 60 years of age, and 116 were >60 years of age. Baseline demographic and disease characteristics were generally well balanced across treatment and age groups (Table 1). However, older patients had longer average disease duration, lower prevalence of extensive disease (pancolitis), more cardiac disorders, and higher frequency of polypharmacy. Exposure to prior immunomodulators, anti-TNF biologics, and non–anti-TNF biologics was lower in older patients.

### Safety in the induction period

The incidences of TEAEs were similar with ozanimod (27.9%−42.3%) and placebo (34.6%−40.4%) across all age groups, with similar incidences in patients receiving ozanimod in older compared with younger patients (Table 2). Rates of serious TEAEs were similar in older and younger patients receiving ozanimod; rates of serious TEAEs in the ozanimod treatment groups across all ages were similar to rates in the placebo group of patients <40 years of age (1.7%−7.2% vs 5.8%, respectively). There were no serious TEAEs in the placebo group of patients >60 years of age during the induction period. Rates of infection were higher in the placebo group (23.1%) than the ozanimod group (cohort 1: 8.5%; cohort 2: 9.3%) in patients >60 years of age. Few TEAEs led to treatment discontinuation across all age groups (2.1%−4.7%).

The most frequent TEAEs in the ozanimod group were nasopharyngitis (1.1%−5.8%), headache (0–5.8%), and anemia (0–5.3%). Diarrhea, nausea, and fatigue were more frequent in those >60 years of age receiving ozanimod than in the younger groups. AEs of special interest, including infection, malignancy, cardiac events, and macular edema, were generally low overall during the induction period and similar between age groups (Table 2). There were no cases of progressive multifocal leukoencephalopathy (PML). One death from acute respiratory distress syndrome due to viral pneumonia occurred during the induction period in an older patient (64 years of age) receiving ozanimod but was deemed unrelated to treatment; the death occurred during a regional outbreak of influenza and the patient had a history of chronic obstructive pulmonary disease.

### Safety in the maintenance period

Rates of TEAEs were higher in patients receiving continuous ozanimod (47.7%−53.6%) than in those who switched to placebo (34.3%−39.2%) in all age groups, with TEAEs occurring slightly more frequently with continuous ozanimod treatment with increasing age (Table 2). Rates of serious TEAEs were similar or slightly higher in patients who switched to placebo (3.8%−8.8%) than those receiving continuous ozanimod (3.6%−6.5%), with few serious TEAEs in patients >60 years of age (only 1 event each in the ozanimod/placebo and ozanimod/ozanimod groups). Rates of TEAEs leading to treatment discontinuation were low and similar between treatment and age groups (0%−3.8%).

The most frequent TEAEs in all ozanimod-treated patients were increased alanine aminotransferase (ALT) (0%−6.4%), arthralgia (1.1%−7.1%), and nasopharyngitis (2.2%−7.1%). The most frequent TEAE in ozanimod-treated patients >60 years of age was peripheral edema (10.7%). AEs of special interest remained low overall during the maintenance period. Rates of infections were higher in patients who received ozanimod than placebo for all age groups (17.9%−25.8% vs 8.8%−15.2%), but there was no increased risk in older patients. There were no malignancies diagnosed during the maintenance period in patients >60 years of age. No events of bradycardia, PML, or deaths occurred during the maintenance period.

### Safety in the OLE

Exposure-adjusted incidence rates (EAIRs) of serious TEAEs (particularly serious infections) and TEAEs leading to treatment discontinuation were highest in patients >60 years of age (11.7 and 3.6, respectively) (Table 3). Of the serious TEAEs, 9 were considered treatment related; 6 occurred in patients <40 years of age, 1 occurred in a patient 40 to 60 years of age, and 2 occurred in patients >60 years of age. Some EAIRs for the most frequent TEAEs (eg, back pain, peripheral edema) were highest in patients >60 years of age, but others (eg, lymphopenia, arthralgia) were not associated with older age. EAIRs of opportunistic infection (eg, herpes zoster), COVID-19, and serious infection increased with age, but most other infections occurred at similar rates across age groups. Patients ≥40 years of age had higher hypertension EAIRs (40–60 years: 3.2; >60 years: 2.6) than those <40 years of age (1.1). EAIRs of other cardiovascular TEAEs were low, and there were few events of complete atrioventricular block and ischemic stroke, which occurred only in patients >60 years of age. Few macular edema events occurred across age groups (1 in each of the 3 age groups). Seventeen malignancies occurred over the ≥4-year period, including 2 events in 2 patients <40 years of age, 9 events in 8 patients 40 to 60 years of age, and 6 events in 6 patients >60 years of age. There were no cases of PML. Three deaths occurred, but none were considered related to ozanimod: 1 sudden death (cause and circumstances unclear) in a patient 57 years of age and 2 deaths in patients >60 years of age (1 due to adenocarcinoma of the pancreas in a patient 62 years of age and 1 due to COVID-19 in a patient 62 years of age).

### ALC and hepatic enzymes

ALC decreased with ozanimod treatment. Decreases from study baseline in ALC were seen at week 5 in patients who received ozanimod during the True North induction period, after which ALC remained stable through the maintenance period and the OLE in patients who received ozanimod; these decreases were similar across age groups (Figures S2 and S3). Reductions in ALC to <500 cells/mm^3^ while receiving ozanimod were common throughout the study and occurred in similar proportions of patients across age groups; few patients had reductions in ALC <200 cells/mm^3^ (Table S1), which were not temporally associated with serious infections.

Elevations in hepatic enzymes (ie, ALT, aspartate aminotransferase, gamma-glutamyl transferase, bilirubin, and alkaline phosphatase) were more frequent with ozanimod treatment and occurred at similar rates across age groups, although elevations in gamma-glutamyl transferase were somewhat more frequent in patients >40 years of age (Table S2). Most ALT and aspartate aminotransferase elevations were transient and resolved without treatment interruption, and no Hy’s law cases or serious hepatic safety events were observed in the induction or maintenance periods or up to the week 190 OLE data cutoff.

### Efficacy in the induction period and maintenance period

**Figure 1.**
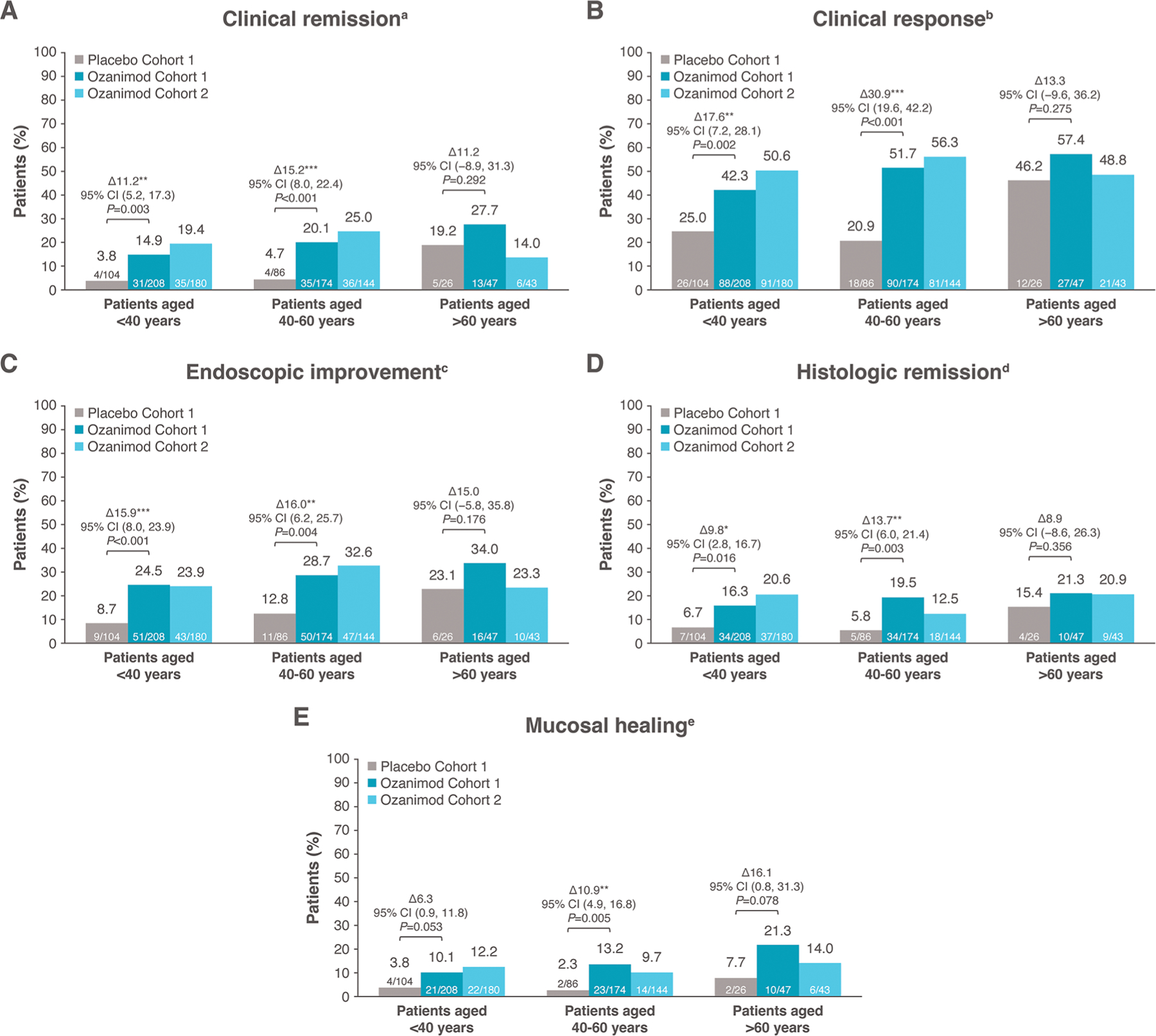
Efficacy during the induction period by age group. (A) Clinical remission. (B) Clinical response. (C) Endoscopic improvement. (D) Histologic remission. (E) Mucosal healing. Treatment differences (Δ), 2-sided Wald confidence intervals (CIs), and *P* values for comparison between ozanimod and placebo (cohort 1) are based on the Cochran-Mantel-Haenszel test, stratified by corticosteroid use at screening and prior anti–tumor necrosis factor use. aRectal bleeding subscore (RBS) = 0, stool frequency subscore ≤1 (and a decrease of ≥1 point from baseline stool frequency subscore), and Mayo endoscopy subscore (MES) ≤1. bReduction from baseline in the 9-point Mayo score of ≥2 points and ≥35%, and a reduction from baseline in RBS of ≥1 point or an absolute RBS ≤1. cMES ≤1 without friability. dGeboes score <2.0. eMES ≤1 and Geboes score <2.0. *P < 0.05. **P < 0.01. ***P < 0.001.

**Figure 2.**
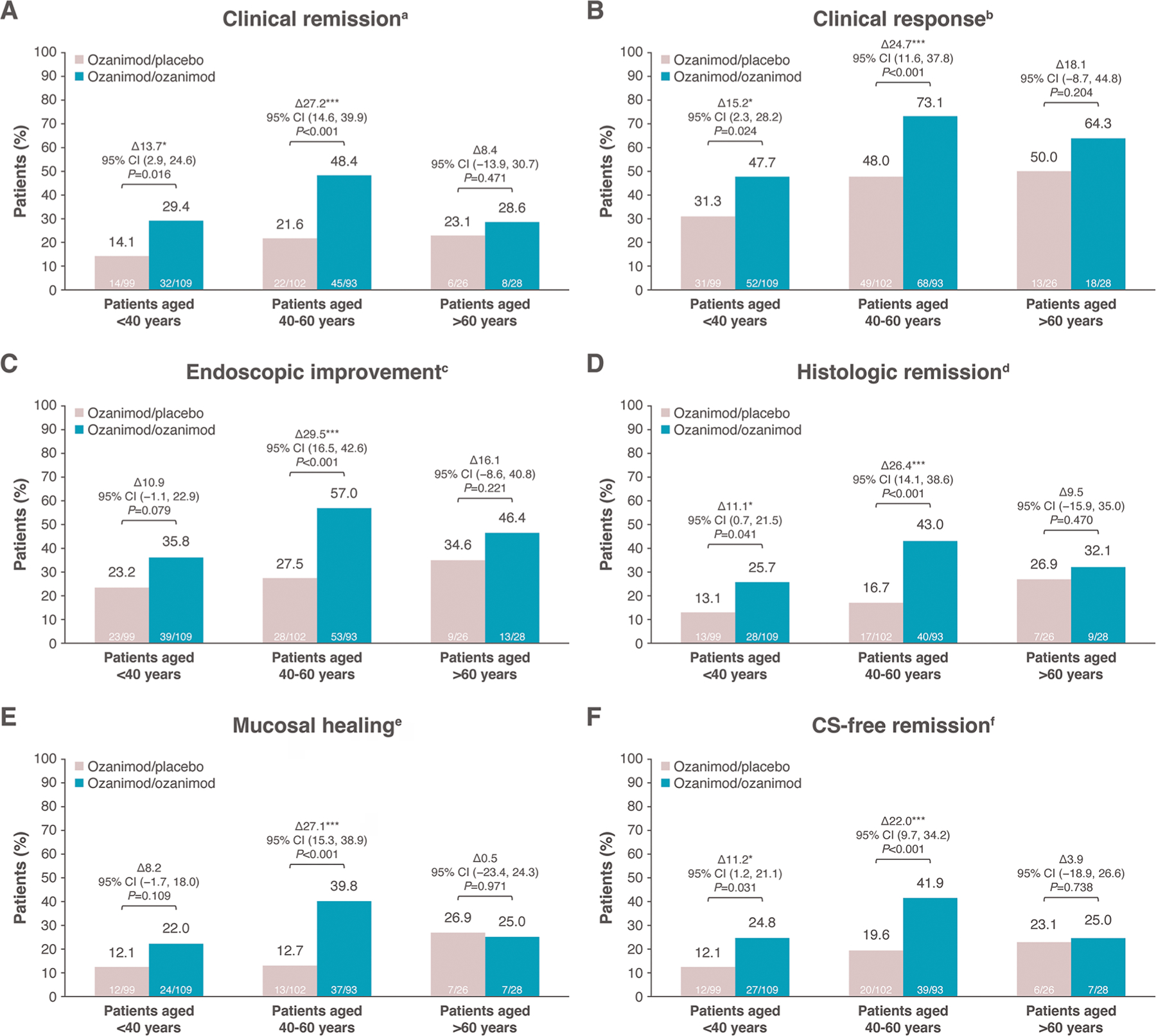
Efficacy during the maintenance period by age group. (A) Clinical remission. (B) Clinical response. (C) Endoscopic improvement. (D) Histologic remission. (E) Mucosal healing. (F) Corticosteroid-free remission. Treatment differences (Δ), 2-sided Wald confidence intervals (CIs), and *P* values for comparison between ozanimod/ozanimod vs ozanimod/placebo are based on the Cochran-Mantel-Haenszel test, stratified by CS use and remission status at week 10. aRectal bleeding subscore (RBS) = 0, stool frequency subscore ≤1 (and a decrease of ≥1 point from baseline stool frequency subscore), and Mayo endoscopy subscore (MES) ≤1. bReduction from baseline in the 9-point Mayo score of ≥2 points and ≥35%, and a reduction from baseline in RBS of ≥1 point or an absolute RBS ≤1. cMES ≤1 without friability. dGeboes score <2.0. eMES ≤1 and Geboes score <2.0. fClinical remission without CS use for ≥12 weeks. *P < 0.05. ***P < 0.001. CS, corticosteroid.

The proportions of patients who achieved clinical remission, clinical response, endoscopic improvement, histologic remission, and mucosal healing at weeks 10 and 52 with ozanimod were generally consistent across age groups (Figures 1A–E and 2A–E). Placebo response rates, however, were highest in those >60 years of age across all efficacy endpoints at weeks 10 and 52; therefore, the adjusted treatment differences for ozanimod vs placebo were not statistically significant (*P* > .05) for all endpoints in patients >60 years of age and were lower for the oldest age group compared with the younger age groups for most endpoints. Similar efficacy rates between ozanimod and placebo were observed for mucosal healing and corticosteroid-free remission at week 52 in the >60 years age group (Figure 2E, F). At week 52, rates of corticosteroid-free remission in patients receiving continuous ozanimod were 24.8% in patients <40 years of age, 41.9% in patients 40 to 60 years of age, and 25.0% in patients >60 years of age (vs 12.1%, 19.6%, and 23.1% with placebo, respectively) (Figure 2F).

### Efficacy in the OLE

**Figure 3.**
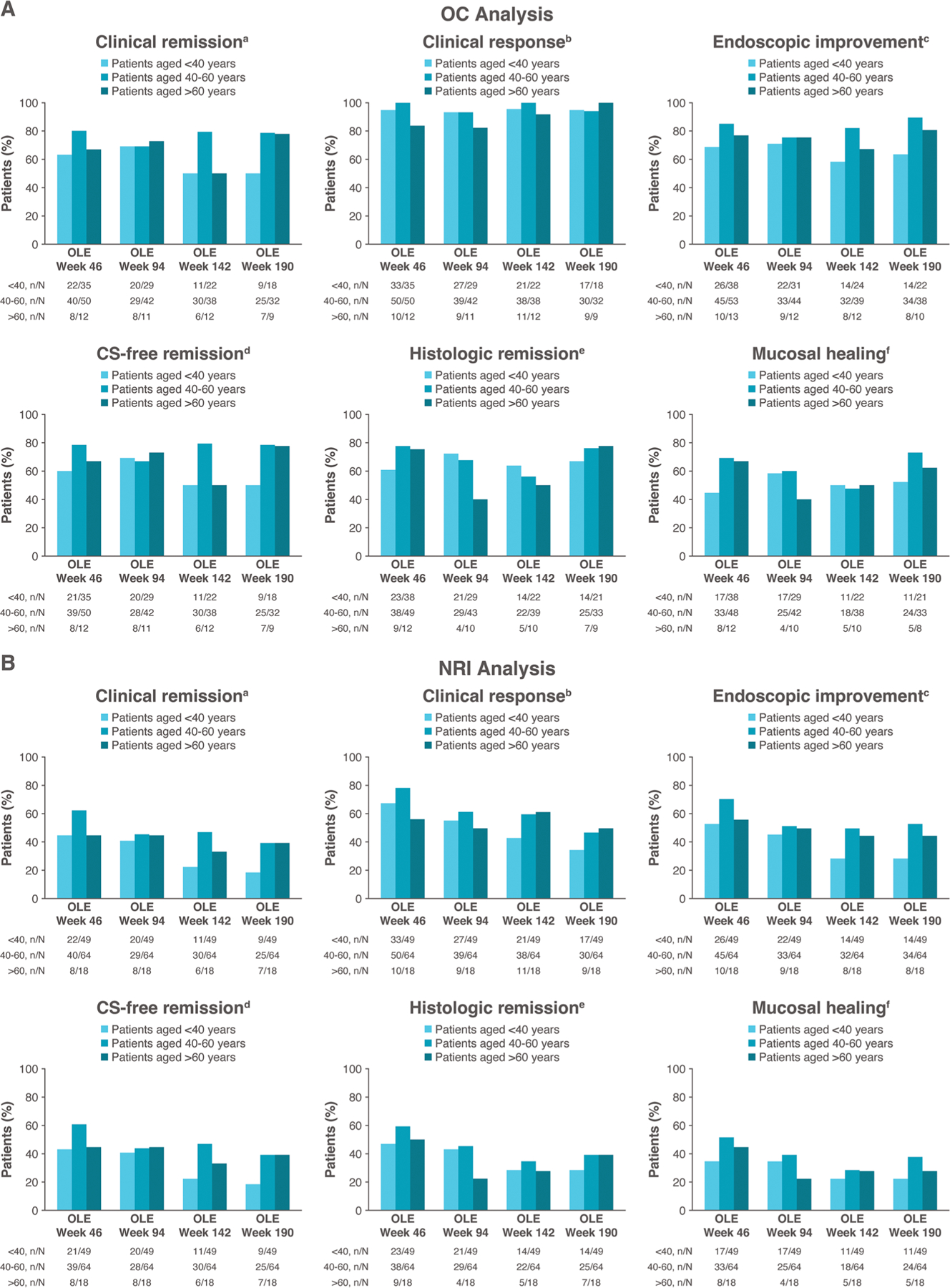
Long-term efficacy during the open-label extension (OLE) by age group in patients receiving continuous ozanimod who entered the OLE in clinical response at True North week 52. (A) Observed case (OC) analysis. (B) Nonresponder imputation (NRI) used for missing data. aRectal bleeding subscore (RBS) = 0, stool frequency subscore ≤1 (and a decrease of ≥1 point from baseline), and Mayo endoscopy subscore (MES) ≤1. bReduction from baseline in 9-point Mayo score of ≥2 points and ≥35%, and reduction from baseline in RBS of ≥1 point or absolute RBS ≤1. cMES ≤1 without friability. dClinical remission while off CS for ≥12 weeks. eGeboes score <2.0. fMES ≤1 and Geboes score <2.0. CS, corticosteroid.

At week 190 and across the OLE, efficacy was generally similar across age groups (Figure 3). At week 190, 50.0% of patients <40 years of age, 78.1% of patients 40 to 60 years of age, and 77.8% of patients >60 years of age were in clinical remission (by observed case analysis; 18.4%, 39.1%, and 38.9%, respectively, by NRI analysis) (Figure 3A, B).

## Discussion

During the first year of treatment in True North, ozanimod safety was generally consistent across age groups. Ozanimod was not associated with higher rates of TEAEs or serious TEAEs in patients >60 years of age. Long-term ozanimod treatment was well tolerated across age groups. Efficacy rates for ozanimod induction and maintenance therapy through 52 weeks were generally consistent across age groups and aligned with the overall population.^27^ Across all age groups, efficacy rates favored ozanimod; however, statistical significance compared with placebo was not achieved in patients >60 years of age.

Age can influence response to therapy. Safety is an important consideration for the treatment of older patients with UC in light of the fact that older patients are more susceptible to AEs and have higher rates of comorbidities and polypharmacy. The incidence of hypertension and serious/opportunistic infections was more frequent in older patients but overall remained low, and other TEAEs were not associated with age. These results are not unexpected, as hypertension and infections are generally more common in older adults with IBD, and more patients >60 years of age had a cardiac disorder at baseline in this study than younger patients.^17,33^ The higher rate of serious infections and herpes zoster infection in older patients may suggest a need to ensure vaccination to prevent these infections. It is recommended to monitor older patients receiving ozanimod treatment for cardiac and hepatic adverse reactions because reduced cardiac and hepatic function is more common in older populations.^25^ Indeed, ∼13% of patients >60 years of age had ≥1 cardiac disorder in this study. Despite this, there were few events of bradycardia, and cardiovascular AEs (other than hypertension) were low in this age group. In addition, although ALC reductions and hepatic enzyme elevations were observed with ozanimod treatment, there was no association with age, and there were no serious hepatic safety events. The incidence of peripheral edema was higher in patients receiving ozanimod in the >60 years age group compared with younger patients during the maintenance period and OLE. Peripheral edema is common with older age, with previous research in the general US population showing 5 times greater odds of peripheral edema in individuals >90 years of age and almost 3 times greater odds in individuals 80 to 89 years of age compared with those 51 to 69 years of age.^34^ In addition, peripheral edema has been reported with S1P receptor modulators approved to treat multiple sclerosis.^35,36^

A recent case series examined cases of PML associated with S1P receptor modulators approved for multiple sclerosis and found that extended exposure to S1P receptor modulators is a risk factor for PML.^37^ Most (53 of 57) cases were associated with fingolimod. Age was also identified as a potential risk factor. Notably, in this case series, only 1 of the 57 reported cases of PML associated with S1P receptor modulators occurred during ozanimod treatment in a patient with multiple sclerosis. This case occurred after 4 years of ozanimod treatment in a 46-year-old woman who had received previous treatment with pegylated interferon β−1a and intramuscular interferon β−1a.^38^ In the present study, there were no cases of PML identified in any age group during the induction period, maintenance period, or OLE.

Some studies have found higher rates of discontinuation of anti-TNF therapy among older patients.^11,39^ In True North, discontinuations due to TEAEs were low and similar between age groups through 52 weeks in the induction and maintenance periods. Discontinuations due to TEAEs were higher in patients >60 years of age in the OLE, although rates were relatively low over a period of 4 years (14.1% in patients >60 years of age vs 7.7% and 6.7% in patients <40 years of age and 40–60 years, respectively).

Treatment differences for efficacy endpoints generally favored ozanimod vs placebo. However, placebo response rates were generally higher in patients >60 years of age than placebo responses in younger patients. Placebo response rates in those >60 years of age were high not only for clinical endpoints, but also for endoscopic and histologic outcomes; mucosal healing and corticosteroid-free remission efficacy rates at week 52 were similar in patients receiving ozanimod and placebo. The cause of these observations is unclear but may potentially be due to the limited sample size and less prior use of corticosteroids, immunomodulators, anti-TNF biologics, and non–anti-TNF biologics at baseline in patients >60 years of age. During the OLE, long-term efficacy of ozanimod was demonstrated across age groups, with the highest efficacy in patients ≥40 years of age.

Our study has several limitations. Subgroup analyses have been limited by small sample size in some groups and a lack of adjustment for imbalances in baseline factors, such as disease severity and prior medication use between groups. Additionally, the exclusion of several comorbid conditions may have resulted in a generally healthier population of older adults in this study compared with the general population. Although our analysis included patients >60 years of age with UC, we did not capture the proportions of those diagnosed with UC at >60 years of age. Future studies would benefit from broader inclusion criteria to better evaluate how coexisting comorbidities and frailty status impact treatment-related safety events and disease-related outcomes. In addition, studies evaluating ozanimod treatment in patients who were diagnosed with UC at an older age (>60 years) would provide further clarity on the effects of ozanimod in older patients. The study is strengthened, however, by an age-matched placebo control group. Due to the high placebo rates seen in older patients in this study, further investigation of ozanimod efficacy in this age group may be warranted.

## Conclusion

Ozanimod safety and efficacy were generally comparable across age groups, although efficacy was not statistically significant when compared with placebo in patients >60 years of age. Due to the unique challenges and risks associated with treating older patients, it is critical to assess drug safety in an older population. To our knowledge, this is the first analysis of ozanimod safety and efficacy in older adults with UC. The results of this study support ozanimod as a safe and tolerable oral treatment option for older patients with UC, including those with underlying cardiovascular disorders.

## Supplementary Material

Supplemental

Supplementary Data

Supplementary data is available at *Inflammatory Bowel Diseases* online.

## Figures and Tables

**Table 1.** Baseline demographic and clinical characteristics by age group.

Characteristic	<40 y (n = 492)			40-60 y (n = 404)			>60 y (n = 116)		
	Cohort 1		Cohort 2	Cohort 1		Cohort 2	Cohort 1		Cohort 2
	Placebo (n = 104)	Ozanimod (n = 208)	Ozanimod (n = 180)	Placebo (n = 86)	Ozanimod (n = 174)	Ozanimod (n = 144)	Placebo (n = 26)	Ozanimod (n = 47)	Ozanimod (n = 43)
**Age, y**	30.3 (19–39)	29.6 (18–39)	30.3 (18–39)	48.9 (40–60)	49.1 (40–60)	50.0 (40–60)	65.6 (61–74)	64.8 (61–72)	65.3 (62–74)
**Male**	65 (62.5)	115 (55.3)	97 (53.9)	62 (72.1)	105 (60.3)	93 (64.6)	16 (61.5)	25 (53.2)	24 (55.8)
**BMI, kg/m** ^2^	23.6 ± 4.2	24.0 ± 5.7	24.7 ± 6.2	26.3 ± 4.2	26.4 ± 5.0	26.8 ± 5.5	27.2 ± 4.6	27.7 ± 4.9	27.5 ± 3.8
**Age at UC diagnosis, y**	25.4 ± 6.0	24.5 ± 6.2	24.6 ± 7.1	40.9 ± 10.2	41.0 ± 8.6	40.8 ± 9.2	56.2 ± 10.7	56.0 ± 9.6	54.4 ± 10.3
**Years since UC diagnosis**	5.0 ± 4.0	5.4 ± 4.6	5.9 ± 5.3	8.2 ± 8.2	8.2 ± 7.1	9.4 ± 7.8	9.4 ± 10.2	9.1 ± 9.9	11.3 ± 10.3
**Extent of disease**									
** Left-sided**	61 (58.7)	118 (56.7)	111 (61.7)	54 (62.8)	115 (66.1)	94 (65.3)	19 (73.1)	35 (74.5)	32 (74.4)
** Extensive (pancolitis)**	43 (41.3)	90 (43.3)	69 (38.3)	32 (37.2)	59 (33.9)	50 (34.7)	7 (26.9)	12 (25.5)	11 (25.6)
**CS use at screening**	41 (39.4)	71 (34.1)	79 (43.9)	27 (31.4)	44 (25.3)	42 (29.2)	8 (30.8)	16 (34.0)	12 (27.9)
**Prior medication use**									
** 5-ASA**	101 (97.1)	199 (95.7)	177 (98.3)	83 (96.5)	173 (99.4)	142 (98.6)	26 (100)	46 (97.9)	43 (100)
** CS**	81 (77.9)	161 (77.4)	145 (80.6)	64 (74.4)	129 (74.1)	108 (75.0)	17 (65.4)	32 (68.1)	33 (76.7)
** IMM**	45 (43.3)	91 (43.8)	87 (48.3)	41 (47.7)	66 (37.9)	63 (43.8)	7 (26.9)	17 (36.2)	16 (37.2)
** Anti-TNF**	36 (34.6)	68 (32.7)	86 (47.8)	23 (26.7)	46 (26.4)	58 (40.3)	6 (23.1)	14 (29.8)	10 (23.3)
** Non–anti-TNF biologic**	24 (23.1)	46 (22.1)	59 (32.8)	14 (16.3)	28 (16.1)	40 (27.8)	6 (23.1)	6 (12.8)	7 (16.3)
**Polypharmacy use (≥4 medications)** ^a^	28 (26.9)	67 (32.2)	77 (42.8)	26 (30.2)	67 (38.5)	65 (45.1)	13 (50.0)	26 (55.3)	27 (62.8)
**FCP, mg/kg**	3425.2 ± 5758.2	2998.5 ± 5253.7	3669.1 ± 6554.6	3391.4 ± 6758.1	2259.7 ± 3777.2	2202.9 ± 3660.0	3659.6 ± 7445.0	1253.9 ± 3114.3	2598.0 ± 5978.2
**CRP, mg/L**	12.1 ± 21.1	7.3 ± 12.1	8.9 ± 12.2	9.3 ± 13.8	8.3 ± 14.5	9.7 ± 15.5	13.1 ± 17.5	9.6 ± 14.9	10.3 ± 12.5
**Total Mayo score** ^b^	9.0 ± 1.4	8.9 ± 1.4	9.3 ± 1.5	8.9 ± 1.3	8.9 ± 1.5	8.9 ± 1.5	8.4 ± 1.4	8.6 ± 1.6	8.5 ± 1.3
**Partial Mayo score** ^c^	6.4 ± 1.2	6.4 ± 1.3	6.7 ± 1.2	6.3 ± 1.1	6.3 ± 1.3	6.3 ± 1.2	5.8 ± 1.2	6.0 ± 1.3	6.0 ± 1.1
**9-point Mayo score** ^d^	6.7 ± 1.2	6.7 ± 1.2	7.0 ± 1.2	6.7 ± 1.1	6.6 ± 1.2	6.6 ± 1.3	6.3 ± 1.1	6.6 ± 1.3	6.3 ± 1.2
**Histologic score**	12.2 ± 4.9	12.7 ± 5.5	13.9 ± 4.4	12.8 ± 4.6	12.6 ± 5.3	14.5 ± 4.4	12.8 ± 5.3	14.0 ± 4.8	14.6 ± 3.8
**RBS**									
** 0**	1 (1.0)	0	0	0	3 (1.7)	1 (0.7)	0	0	0
** 1**	34 (32.7)	79 (38.0)	55 (30.6)	43 (50.0)	71 (40.8)	67 (46.5)	16 (61.5)	20 (42.6)	25 (58.1)
** 2**	67 (64.4)	109 (52.4)	102 (56.7)	37 (43.0)	88 (50.6)	73 (50.7)	10 (38.5)	26 (55.3)	17 (39.5)
** 3**	2 (1.9)	20 (9.6)	23 (12.8)	6 (7.0)	12 (6.9)	3 (2.1)	0	1 (2.1)	1 (2.3)
**SFS**									
** 0**	0	0	0	0	0	0	0	0	0
** 1**	12 (11.5)	32 (15.4)	15 (8.3)	7 (8.1)	20 (11.5)	21 (14.6)	4 (15.4)	5 (10.6)	7 (16.3)
** 2**	37 (35.6)	64 (30.8)	52 (28.9)	31 (36.0)	62 (35.6)	42 (29.2)	9 (34.6)	19 (40.4)	17 (39.5)
** 3**	55 (52.9)	112 (53.8)	113 (62.8)	48 (55.8)	92 (52.9)	81 (56.3)	13 (50.0)	23 (48.9)	19 (44.2)
**PGA subscore**									
** 0**	0	0	1 (0.6)	0	0	0	1 (3.8)	0	0
** 1**	2 (1.9)	11 (5.3)	2 (1.1)	2 (2.3)	6 (3.4)	4 (2.8)	0	5 (10.6)	1 (2.3)
** 2**	72 (69.2)	131 (63.0)	112 (62.2)	60 (69.8)	121 (69.5)	88 (61.1)	21 (80.8)	34 (72.3)	31 (72.1)
** 3**	30 (28.8)	66 (31.7)	65 (36.1)	24 (27.9)	47 (27.0)	52 (36.1)	4 (15.4)	8 (17.0)	11 (25.6)
**MES**									
** 0**	0	0	0	0	0	0	0	0	0
** 1**	0	0	0	0	0	0	0	0	0
** 2**	42 (40.4)	87 (41.8)	70 (38.9)	33 (38.4)	73 (42.0)	50 (34.7)	11 (42.3)	19 (40.4)	18 (41.9)
** 3**	62 (59.6)	121 (58.2)	110 (61.1)	53 (61.6)	101 (58.0)	94 (65.3)	15 (57.7)	28 (59.6)	25 (58.1)
**≥1 Cardiac disorder** ^e^	1 (1.0)	9 (4.3)	7 (3.9)	5 (5.8)	13 (7.5)	13 (9.0)	6 (12.8)	6 (23.1)	6 (14.0)

Values are mean (range), n (%), or mean ± SD.

Abbreviations: 5-ASA, 5-aminosalicylic acid; BMI, body mass index; CRP, C-reactive protein; CS, corticosteroid; FCP, fecal calprotectin; IMM, immunomodulator; MES, Mayo endoscopy subscore; PGA, Physician Global Assessment; RBS, rectal bleeding subscore; SFS, stool frequency subscore; TNF, tumor necrosis factor; UC, ulcerative colitis.

a Polypharmacy use was defined as patients who received ≥4 prior or ongoing medications of any kind with no stop date.

b Total Mayo score is the sum of the RBS, SFS, PGA subscore, and MES.

c Partial Mayo score is the sum of RBS, SFS, and PGA subscore.

d The 9-point Mayo score is the sum of RBS, SFS, and MES.

e As identified from the patient’s medical history. Cardiac disorders included angina pectoris, arrhythmia, atrial fibrillation, atrial flutter, atrioventricular block first degree, bundle branch block left, bundle branch block right, cardiac failure, cardiac failure chronic, cardiomyopathy, coronary artery disease, diastolic dysfunction, extrasystoles, ischemic cardiomyopathy, left atrial enlargement, metabolic cardiomyopathy, mitral valve incompetence, mitral valve prolapse, myocardial infarction, myocardial ischemia, myocarditis, palpitations, paroxysmal arrhythmia, pericardial effusion, pericarditis, rheumatic heart disease, sinus arrhythmia, sinus tachycardia, supraventricular extrasystoles, supraventricular tachycardia, tachycardia, and Wolff-Parkinson-White syndrome.

**Table 2.** TEAEs during the induction period and maintenance period by age group.

Induction period									
TEAEs	<40 y (n = 492)			40-60 y (n = 404)			>60 y (n = 116)		
	Cohort 1		Cohort 2	Cohort 1		Cohort 2	Cohort 1		Cohort 2
	Placebo	Ozanimod	Ozanimod	Placebo	Ozanimod	Ozanimod	Placebo	Ozanimod	Ozanimod
	(n = 104)	(n = 208)	(n = 180)	(n = 86)	(n = 174)	(n = 144)	(n = 26)	(n = 47)	(n = 43)
**Any TEAE**	42 (40.4)	88 (42.3)	75 (41.7)	31 (36.0)	66 (37.9)	59 (41.0)	9 (34.6)	18 (38.3)	12 (27.9)
**Serious TEAE**	6 (5.8)	11 (5.3)	13 (7.2)	1 (1.2)	3 (1.7)	8 (5.6)	0	3 (6.4)	2 (4.7)
**TEAE leading to treatment discontinuation**	3 (2.9)	7 (3.4)	8 (4.4)	3 (3.5)	6 (3.4)	4 (2.8)	1 (3.8)	1 (2.1)	2 (4.7)
**Most frequent TEAEs (>5%)**									
** Nasopharyngitis**	1 (1.0)	12 (5.8)	7 (3.9)	0	2 (1.1)	2 (1.4)	2 (7.7)	1 (2.1)	1 (2.3)
** Headache**	4 (3.8)	12 (5.8)	4 (2.2)	0	2 (1.1)	4 (2.8)	0	0	2 (4.7)
** Anemia**	10 (9.6)	11 (5.3)	9 (5.0)	2 (2.3)	7 (4.0)	6 (4.2)	0	0	1 (2.3)
** Nausea**	2 (1.9)	8 (3.8)	3 (1.7)	1 (1.2)	1 (0.6)	0	0	3 (6.4)	0
** Diarrhea**	1 (1.0)	2 (1.0)	0	0	1 (0.6)	0	1 (3.8)	3 (6.4)	0
** Fatigue**	1 (1.0)	1 (0.5)	0	0	3 (1.7)	1 (0.7)	0	3 (6.4)	0
**Infections**	13 (12.5)	27 (13.0)	25 (13.9)	6 (7.0)	15 (8.6)	17 (11.8)	6 (23.1)	4 (8.5)	4 (9.3)
** Herpes zoster** ^a^	0	0	1 (0.6)	0	2 (1.1)	0	0	0	0
** Serious infection**	1 (1.0)	1 (0.5)	2 (1.1)	0	2 (1.1)	2 (1.4)	0	1 (2.1)	2 (4.7)
**Malignancy**									
** Cervix carcinoma stage 0**	0	0	1 (0.6)	0	0	0	0	0	0
** Basal cell carcinoma**	0	0	0	0	0	1 (0.7)	0	0	0
**Cardiovascular disorders**									
** Hypertension**	0	3 (1.4)	3 (1.7)	0	2 (1.1)	2 (1.4)	0	1 (2.1)	2 (4.7)
** Bradycardia**	0	1 (0.5)	1 (0.6)	0	0	0	0	1 (2.1)	2 (4.7)
** Ischemic stroke**	0	0	0	0	0	0	0	1 (2.1)	0
**Macular edema**	0	0	0	0	1 (0.6)	1 (0.7)	0	0	0

**Maintenance period**									

**TEAE**			**<40 y (n = 208)**		**40-60 y (n = 195)**		**>60 y (n = 54)**		
			**Ozanimod/placebo (n = 99)**	**Ozanimod/ozanimod (n = 109)**	**Ozanimod/placebo (n = 102)**	**Ozanimod/ozanimod (n = 93)**	**Ozanimod/ placebo (n = 26)**		**Ozanimod/ozanimod (n = 28)**

**Any TEAE**			34 (34.3)	52 (47.7)	40 (39.2)	46 (49.5)	9 (34.6)		15 (53.6)
**Serious TEAE**			8 (8.1)	5 (4.6)	9 (8.8)	6 (6.5)	1 (3.8)		1 (3.6)
**TEAE leading to treatment discontinuation**			3 (3.0)	2 (1.8)	2 (2.0)	0	1 (3.8)		1 (3.6)
**Most frequent TEAEs (>5%)**									
** ALT increased**			0	7 (6.4)	1 (1.0)	4 (4.3)	0		0
** Arthralgia**			0	4 (3.7)	6 (5.9)	1 (1.1)	0		2 (7.1)
** Nasopharyngitis**			2 (2.0)	3 (2.8)	2 (2.0)	2 (2.2)	0		2 (7.1)
** Ulcerative colitis**			5 (5.1)	1 (0.9)	5 (4.9)	0	0		0
** Peripheral edema**			0	1 (0.9)	0	2 (2.2)	0		3 (10.7)
** Urinary tract infection**			1 (1.0)	0	1 (1.0)	0	2 (7.7)		0
** GGT increased**			0	1 (0.9)	1 (1.0)	4 (4.3)	0		2 (7.1)
** Alopecia**			1 (1.0)	1 (0.9)	1 (1.0)	0	0		2 (7.1)
**Infections**			15 (15.2)	24 (22.0)	9 (8.8)	24 (25.8)	3 (11.5)		5 (17.9)
** Herpes zoster** ^a^			1 (1.0)	1 (0.9)	0	3 (3.2)	0		1 (3.6)
** Serious infection**			3 (3.0)	1 (0.9)	1 (1.0)	1 (1.1)	0		0
**Malignancy**									
** Adenocarcinoma of colon**			1 (1.0)	0	0	0	0		0
** Basal cell carcinoma**			0	0	0	1 (1.1)	0		0
** Breast cancer**			0	0	1 (1.0)	0	0		0
** Rectal adenocarcinoma**			0	0	0	1 (1.1)	0		0
**Cardiovascular disorders**									
** Hypertension**			0	1 (0.9)	3 (2.9)	3 (3.2)	0		0
** Arteriosclerosis**			0	0	0	1 (1.1)	0		0
**Macular edema**			0	0	0	0	0		1 (3.6)

Values are n (%).

Abbreviations: ALT, alanine aminotransferase; GGT, gamma-glutamyl transferase; TEAE, treatment-emergent adverse event; VZV, varicella zoster virus.

a Per study eligibility criteria, patients must have had documentation of positive VZV immunoglobulin antibody status or complete VZV vaccination ≥30 days prior to randomization in True North.

**Table 3.** TEAEs During True North and the OLE in patients who entered the OLE.

TEAE	<40 y (n = 405)		40-60 y (n = 326)		>60 y (n = 92)	
	n (%)	EAIR^a^	n (%)	EAIR^a^	n (%)	EAIR^a^
**Any TEAE**	332 (82.0)	98.5	271 (83.1)	72.9	84 (91.3)	85.3
**Serious TEAE**	71 (17.5)	7.0	58 (17.8)	5.4	34 (37.0)	11.7
**TEAE leading to treatment**	31 (7.7)	2.8	22 (6.7)	1.9	13 (14.1)	3.6
**discontinuation**						
**Most frequent TEAEs (>7%)**						
** Lymphopenia**	69 (17.0)	7.0	54 (16.6)	5.1	15 (16.3)	4.7
** Anemia**	52 (12.8)	5.2	33 (10.1)	3.0	5 (5.4)	1.4
** Nasopharyngitis**	51 (12.6)	5.0	29 (8.9)	2.6	9 (9.8)	2.7
** Lymphocyte count decreased**	47 (11.6)	4.5	32 (9.8)	2.9	10 (10.9)	3.0
** ALT increased**	39 (9.6)	3.8	35 (10.7)	3.2	4 (4.3)	1.1
** Headache**	37 (9.1)	3.6	22 (6.7)	2.0	6 (6.5)	1.7
** COVID-19**	36 (8.9)	3.4	50 (15.3)	4.5	17 (18.5)	5.0
** Arthralgia**	34 (8.4)	3.2	33 (10.1)	3.0	9 (9.8)	2.6
** Upper respiratory tract infection**	31 (7.7)	3.0	22 (6.7)	2.0	5 (5.4)	1.5
** UC exacerbation**	31 (7.7)	2.8	11 (3.4)	0.9	4 (4.3)	1.1
** GGT increased**	22 (5.4)	2.1	30 (9.2)	2.7	5 (5.4)	1.4
** Back pain**	12 (3.0)	1.1	14 (4.3)	1.2	12 (13.0)	3.6
** Peripheral edema**	8 (2.0)	0.7	10 (3.1)	0.9	7 (7.6)	2.0
**Infections**	179 (44.2)	25.0	153 (46.9)	19.8	49 (53.3)	21.3
** Herpes zoster** ^b^	10 (2.5)	0.9	13 (4.0)	1.1	8 (8.7)	2.4
** Serious infection**	15 (3.7)	1.4	18 (5.5)	1.6	12 (13.0)	3.5
**Malignancy**						
** Adenocarcinoma of colon**	1 (0.2)	0.1	0	0	0	0
** Basal cell carcinoma**	1 (0.2)	0.1	5 (1.5)	0.4	1 (1.1)	0.3
** Breast cancer**	0	0	0	0	1 (1.1)	0.3
** Rectal adenocarcinoma**	0	0	0	0	0	0
** B-cell lymphoma**	0	0	1 (0.3)	0.1	0	0
** Rectal cancer stage II**	0	0	1 (0.3)	0.1	0	0
** Rectal adenocarcinoma**	0	0	1 (0.3)	0.1	0	0
** Squamous cell carcinoma of skin**	0	0	1 (0.3)	0.1	0	0
** Adenocarcinoma pancreas**	0	0	0	0	1 (1.1)	0.3
** Follicular lymphoma stage IV**	0	0	0	0	1 (1.1)	0.3
** Lung neoplasm malignant**	0	0	0	0	1 (1.1)	0.3
** Prostate cancer**	0	0	0	0	1 (1.1)	0.3
**Cardiovascular disorders**						
** Hypertension**	12 (3.0)	1.1	35 (10.7)	3.2	9 (9.8)	2.6
** Bradycardia**	2 (0.5)	0.2	0	0	1 (1.1)	0.3
** Complete AV block**	0	0	0	0	1 (1.1)	0.3
** Myocardial ischemia**	0	0	2 (0.6)	0.2	1 (1.1)	0.3
** Ischemic stroke**	0	0	0	0	3 (3.3)	0.8
** Pulmonary embolism**	1 (0.2)	0.1	1 (0.3)	0.1	1 (1.1)	0.3
** Deep vein thrombosis**	1 (0.2)	0.1	1 (0.3)	0.1	1 (1.1)	0.3
**Ophthalmic**						
** Macular edema**	1 (0.2)	0.1	1 (0.3)	0.1	1 (1.1)	0.3
** Cystoid macular edema**	1 (0.2)	0.1	0	0	2 (2.2)	0.6

Abbreviations: ALT, alanine aminotransferase; AV, atrioventricular; EAIR, exposure-adjusted incidence rate; GGT, gamma-glutamyl transferase; OLE, open-label extension; TEAE, treatment-emergent adverse event; UC, ulcerative colitis; VZV, varicella zoster virus.

a EAIRs expressed per 100 patient-years.

b Per study eligibility criteria, patients must have had documentation of positive VZV immunoglobulin antibody status or complete VZV vaccination ≥30 days prior to randomization in True North.

## Data Availability

Bristol Myers Squibb policy on data sharing may be found at https://www.bms.com/researchers-and-partners/independent-research/data-sharing-request-process.html. De-identified individual patient data will not be shared.

## Key Messages

### What is already known?

Ozanimod is an oral small molecule and selective sphingosine 1-phosphate receptor modulator approved for the treatment of patients with moderately to severely active ulcerative colitis (UC).

### What is new here?

We show ozanimod safety and efficacy were generally similar across age groups in patients with UC. However, efficacy was not statistically significant in patients >60 years of age and the incidence of serious infections, albeit low, increased with age during the open-label extension.

### How can this study help patient care?

Our results support ozanimod as a safe and tolerable oral treatment option for older patients with UC.
