# Supplementary material for: Safety and Efficacy of Ozanimod in Patients With Moderately to Severely Active Ulcerative Colitis Stratified by Age
Source: Inflamm Bowel Dis. Author manuscript; Available in PMC 2026 Jan 13. (PMC12794806; doi:10.1093/ibd/izaf258)
Supplement: Supplemental [file NIHMS2127445-supplement-Supplemental.docx]

**Supplementary Information**

**Supplementary Table S1. ALC Reductions During True North and the OLE by Age Group**

| **Induction Period** | | | | | | | | | | | | |
| --- | --- | --- | --- | --- | --- | --- | --- | --- | --- | --- | --- | --- |
|  | **Patients aged <40 years**  **(n=492)** | | | | **Patients aged 40–60 years**  **(n=404)** | | | | **Patients aged >60 years**  **(n=116)** | | | |
|  | **Cohort 1** | | | **Cohort 2** | **Cohort 1** | | | **Cohort 2** | **Cohort 1** | | | **Cohort 2** |
|  | **Placebo**  **(n=104)** | **Ozanimod**  **(n=208)** | | **Ozanimod**  **(n=180)** | **Placebo**  **(n=86)** | **Ozanimod**  **(n=174)** | | **Ozanimod**  **(n=144)** | **Placebo**  **(n=26)** | **Ozanimod**  **(n=47)** | | **Ozanimod**  **(n=43)** |
| Patients with an assessment, n | 100 | 207 | | 176 | 84 | 169 | | 141 | 25 | 46 | | 42 |
| ALC <200 cells/µl, n (%) | 0 | 4 (1.9) | | 2 (1.1) | 0 | 2 (1.2) | | 1 (0.7) | 0 | 0 | | 0 |
| ALC <500 cells/µl, n (%) | 0 | 63 (30.4) | | 59 (33.5) | 0 | 34 (20.1) | | 43 (30.5) | 0 | 12 (26.1) | | 10 (23.8) |
| **Maintenance Period** | | | | | | | | | | | | |
|  | **Patients aged <40 years**  **(n=208)** | | | | **Patients aged 40–60 years**  **(n=195)** | | | | **Patients aged >60 years**  **(n=54)** | | | |
|  | **Ozanimod/**  **placebo**  **(n=99)** | | **Ozanimod/**  **ozanimod**  **(n=109)** | | **Ozanimod/**  **placebo**  **(n=102)** | | **Ozanimod/**  **ozanimod**  **(n=93)** | | **Ozanimod/**  **placebo**  **(n=26)** | | **Ozanimod/**  **ozanimod**  **(n=28)** | |
| Patients with an assessment, n | 93 | | 104 | | 98 | | 92 | | 26 | | 26 | |
| ALC <200  cells/µl, n (%) | 0 | | 2 (1.9) | | 0 | | 3 (3.3) | | 0 | | 0 | |
| ALC <500  cells/µl, n (%) | 1 (1.1) | | 50 (48.1) | | 1 (1.0) | | 35 (38.0) | | 0 | | 12 (46.2) | |
| **OLE^a^** | | | | | | | | | | | | |
|  | **Patients aged <40 years**  **(n=405)** | | | | **Patients aged 40–60 years**  **(n=326)** | | | | **Patients aged >60 years**  **(n=92)** | | | |
| Patients with an assessment, n | 403 | | | | 324 | | | | 90 | | | |
| ALC <200 cells/µl, n (%) | 28 (6.9) | | | | 21 (6.5) | | | | 5 (5.6) | | | |
| ALC <500 cells/µl, n (%) | 237 (58.8) | | | | 176 (54.3) | | | | 54 (60.0) | | | |

^a^Includes all patients who entered the OLE.

ALC, absolute lymphocyte count; OLE, open-label extension.

**Supplementary Table S2. Hepatic Enzyme Elevations During True North and the OLE by Age Group**

| **Induction Period** | | | | | | | | | | | | |
| --- | --- | --- | --- | --- | --- | --- | --- | --- | --- | --- | --- | --- |
|  | **Patients aged <40 years**  **(n=492)** | | | | **Patients aged 40–60 years**  **(n=404)** | | | | **Patients aged >60 years**  **(n=116)** | | | |
|  | **Cohort 1** | | | **Cohort 2** | **Cohort 1** | | | **Cohort 2** | **Cohort 1** | | | **Cohort 2** |
|  | **Placebo**  **(n=104)** | **Ozanimod**  **(n=208)** | | **Ozanimod**  **(n=180)** | **Placebo**  **(n=86)** | **Ozanimod**  **(n=174)** | | **Ozanimod**  **(n=144)** | **Placebo**  **(n=26)** | **Ozanimod**  **(n=47)** | | **Ozanimod**  **(n=43)** |
| ALT |  |  | |  |  |  | |  |  |  | |  |
| N | 100 | 208 | | 175 | 84 | 169 | | 141 | 25 | 46 | | 42 |
| ≥3 × ULN, n (%) | 0 | 6 (2.9) | | 2 (1.1) | 0 | 4 (2.4) | | 3 (2.1) | 0 | 0 | | 1 (2.4) |
| ≥5 × ULN, n (%) | 0 | 3 (1.4) | | 0 | 0 | 0 | | 1 (0.7) | 0 | 0 | | 0 |
| AST |  |  | |  |  |  | |  |  |  | |  |
| N | 100 | 208 | | 175 | 84 | 169 | | 141 | 25 | 46 | | 42 |
| ≥3 × ULN, n (%) | 0 | 3 (1.4) | | 1 (0.6) | 0 | 2 (1.2) | | 1 (0.7) | 0 | 0 | | 0 |
| ≥5 × ULN, n (%) | 0 | 2 (1.0) | | 0 | 0 | 1 (0.6) | | 0 | 0 | 0 | | 0 |
| GGT |  |  | |  |  |  | |  |  |  | |  |
| N | 100 | 208 | | 176 | 84 | 169 | | 141 | 25 | 46 | | 42 |
| ≥3 × ULN, n (%) | 0 | 5 (2.4) | | 5 (2.8) | 3 (3.6) | 9 (5.3) | | 10 (7.1) | 2 (8.0) | 1 (2.2) | | 1 (2.4) |
| ≥5 × ULN, n (%) | 0 | 4 (1.9) | | 5 (2.8) | 1 (1.2) | 3 (1.8) | | 3 (2.1) | 1 (4.0) | 0 | | 1 (2.4) |
| Bilirubin |  |  | |  |  |  | |  |  |  | |  |
| N | 100 | 208 | | 176 | 84 | 169 | | 141 | 25 | 46 | | 42 |
| ≥3 × ULN, n (%) | 0 | 0 | | 0 | 0 | 0 | | 0 | 0 | 0 | | 0 |
| ≥5 × ULN, n (%) | 0 | 0 | | 0 | 0 | 0 | | 0 | 0 | 0 | | 0 |
| ALP |  |  | |  |  |  | |  |  |  | |  |
| N | 100 | 208 | | 176 | 84 | 169 | | 141 | 25 | 46 | | 42 |
| ≥3 × ULN, n (%) | 0 | 1 (0.5) | | 0 | 0 | 1 (0.6) | | 0 | 1 (4.0) | 0 | | 0 |
| ≥5 × ULN, n (%) | 0 | 0 | | 0 | 0 | 0 | | 0 | 0 | 0 | | 0 |
| **Maintenance Period** | | | | | | | | | | | | |
|  | **Patients aged <40 years**  **(n=208)** | | | | **Patients aged 40–60 years**  **(n=195)** | | | | **Patients aged >60 years**  **(n=54)** | | | |
|  | **Ozanimod/**  **placebo**  **(n=99)** | | **Ozanimod/**  **ozanimod**  **(n=109)** | | **Ozanimod/**  **placebo**  **(n=102)** | | **Ozanimod/**  **ozanimod**  **(n=93)** | | **Ozanimod/**  **placebo**  **(n=26)** | | **Ozanimod/**  **ozanimod**  **(n=28)** | |
| ALT |  | |  | |  | |  | |  | |  | |
| N | 94 | | 104 | | 98 | | 92 | | 26 | | 26 | |
| ≥3 × ULN, n (%) | 0 | | 2 (1.9) | | 0 | | 1 (1.1) | | 0 | | 0 | |
| ≥5 × ULN, n (%) | 0 | | 1 (1.0) | | 0 | | 0 | | 0 | | 0 | |
| AST |  | |  | |  | |  | |  | |  | |
| N | 94 | | 104 | | 98 | | 92 | | 26 | | 26 | |
| ≥3 × ULN, n (%) | 0 | | 2 (1.9) | | 1 (1.0) | | 0 | | 0 | | 0 | |
| ≥5 × ULN, n (%) | 0 | | 0 | | 0 | | 0 | | 0 | | 0 | |
| GGT |  | |  | |  | |  | |  | |  | |
| N | 94 | | 104 | | 98 | | 92 | | 26 | | 26 | |
| ≥3 × ULN, n (%) | 0 | | 5 (4.8) | | 5 (5.1) | | 13 (14.1) | | 1 (3.8) | | 3 (11.5) | |
| ≥5 × ULN, n (%) | 0 | | 1 (1.0) | | 2 (2.0) | | 6 (6.5) | | 1 (3.8) | | 2 (7.7) | |
| Bilirubin |  | |  | |  | |  | |  | |  | |
| N | 94 | | 104 | | 98 | | 92 | | 26 | | 26 | |
| ≥3 × ULN, n (%) | 0 | | 0 | | 1 (1.0) | | 0 | | 0 | | 0 | |
| ≥5 × ULN, n (%) | 0 | | 0 | | 0 | | 0 | | 0 | | 0 | |
| ALP |  | |  | |  | |  | |  | |  | |
| N | 94 | | 104 | | 98 | | 92 | | 26 | | 26 | |
| ≥3 × ULN, n (%) | 0 | | 0 | | 1 (1.0) | | 0 | | 0 | | 0 | |
| ≥5 × ULN, n (%) | 0 | | 0 | | 1 (1.0) | | 0 | | 0 | | 0 | |
| **OLE^a^** | | | | | | | | | | | | |
|  | **Patients aged <40 years**  **(n=405)** | | | | **Patients aged 40–60 years**  **(n=326)** | | | | **Patients aged >60 years**  **(n=92)** | | | |
| ALT |  | | | |  | | | |  | | | |
| N | 403 | | | | 324 | | | | 90 | | | |
| ≥3 × ULN, n (%) | 30 (7.4) | | | | 24 (7.4) | | | | 3 (3.3) | | | |
| ≥5 × ULN, n (%) | 10 (2.5) | | | | 5 (1.5) | | | | 2 (2.2) | | | |
| AST |  | | | |  | | | |  | | | |
| N | 403 | | | | 324 | | | | 90 | | | |
| ≥3 × ULN, n (%) | 17 (4.2) | | | | 7 (2.2) | | | | 2 (2.2) | | | |
| ≥5 × ULN, n (%) | 4 (1.0) | | | | 1 (0.3) | | | | 1 (1.1) | | | |
| GGT |  | | | |  | | | |  | | | |
| N | 403 | | | | 324 | | | | 90 | | | |
| ≥3 × ULN, n (%) | 42 (10.4) | | | | 67 (20.7) | | | | 13 (14.4) | | | |
| ≥5 × ULN, n (%) | 14 (3.5) | | | | 28 (8.6) | | | | 8 (8.9) | | | |
| Bilirubin |  | | | |  | | | |  | | | |
| N | 403 | | | | 324 | | | | 90 | | | |
| ≥3 × ULN, n (%) | 0 | | | | 0 | | | | 0 | | | |
| ≥5 × ULN, n (%) | 0 | | | | 0 | | | | 0 | | | |
| ALP |  | | | |  | | | |  | | | |
| N | 403 | | | | 324 | | | | 90 | | | |
| ≥3 × ULN, n (%) | 4 (1.0) | | | | 6 (1.9) | | | | 1 (1.1) | | | |
| ≥5 × ULN, n (%) | 0 | | | | 0 | | | | 0 | | | |

^a^Includes all patients who entered the OLE.

ALP, alkaline phosphatase; ALT, alanine aminotransferase; AST, aspartate aminotransferase; GGT, gamma-glutamyl transferase; OLE, open-label extension; ULN, upper limit of normal.

**Supplementary Figure S1.** Study design. ^a^Patients stratified by prior tumour necrosis factor inhibitor exposure (yes/no) and corticosteroid use (yes/no) at screening. ^b^Reduction in 3-component Mayo score of ≥2 points and ≥35% or reduction in 4-component Mayo score of ≥3 points and ≥30%, and reduction in RBS of ≥1 point or absolute RBS of ≤1 point. ^c^Disease relapse was defined as partial Mayo score increase ≥2 points versus the Week 10 score and absolute score ≥4 points, Mayo endoscopy subscore of ≥2 points, and exclusion of other causes of an increase in disease activity unrelated to underlying ulcerative colitis. OLE, open-label extension; RBS, rectal bleeding subscore.

**
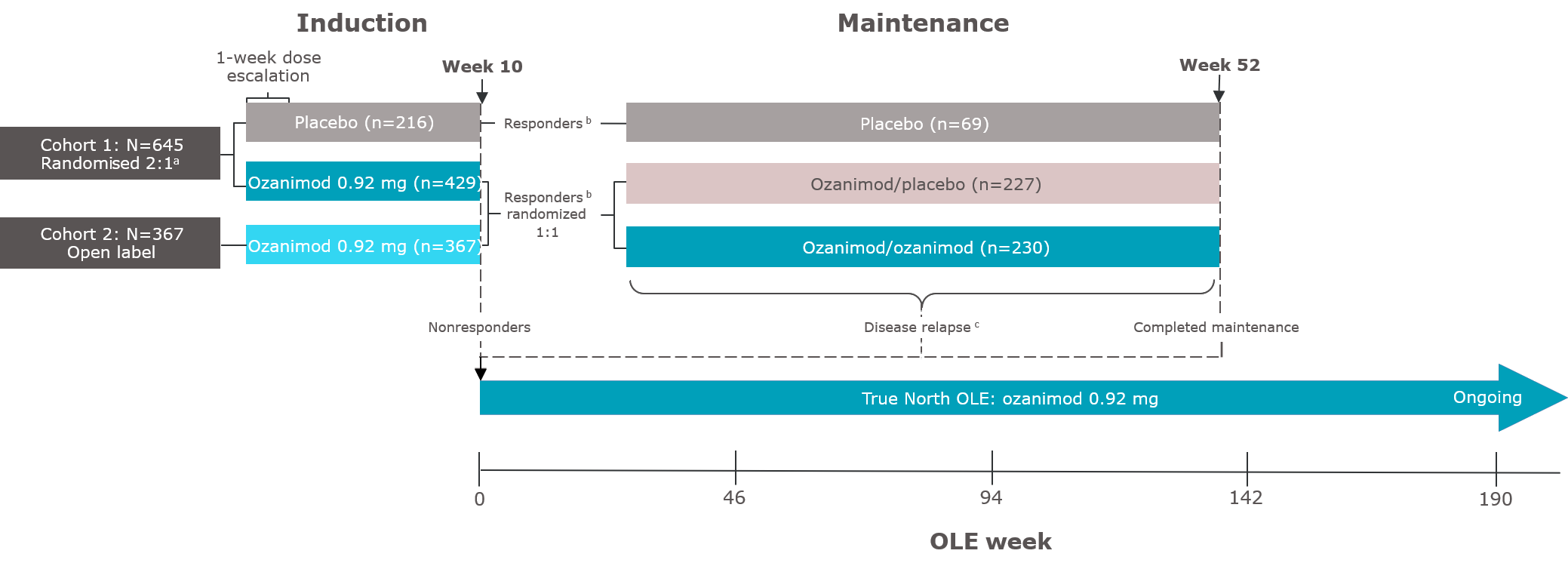
**

**Supplementary Figure S2.** Mean ALC over time during True North by age group. (A) Induction period. (B) Maintenance period. ^a^Baseline defined as the last measurement collected on or prior to the first dose of study drug (IP baseline). ALC, absolute lymphocyte count.

**
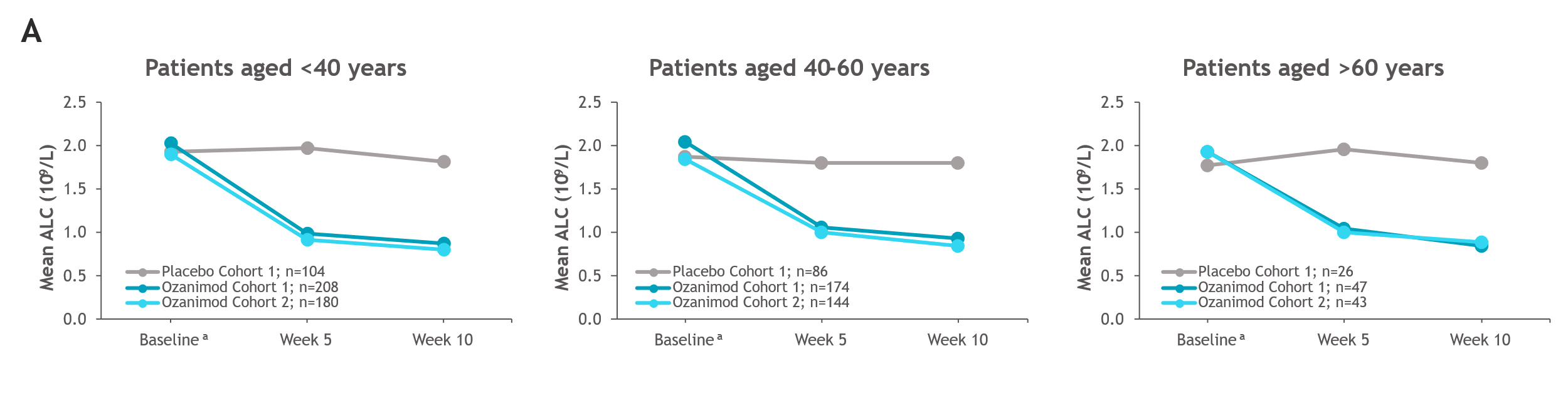
**

**
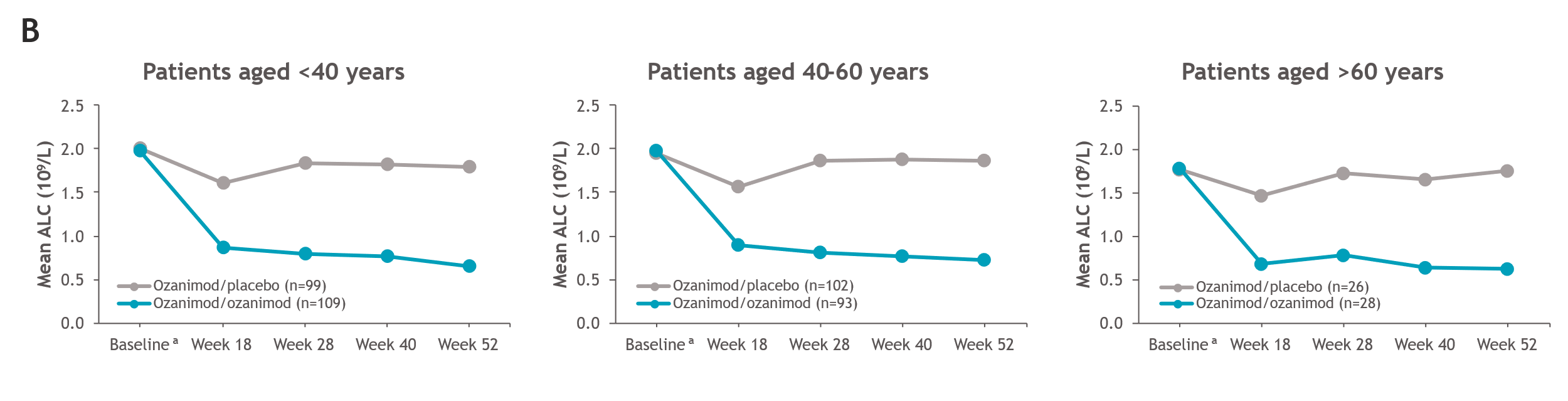
**

**Supplementary** **Figure S3.** Mean ALC over time during the OLE by age group. ^a^Baseline defined as the last measurement collected on or prior to the first doses of study drug (induction period baseline). ALC, absolute lymphocyte count; OLE, open-label extension.

**
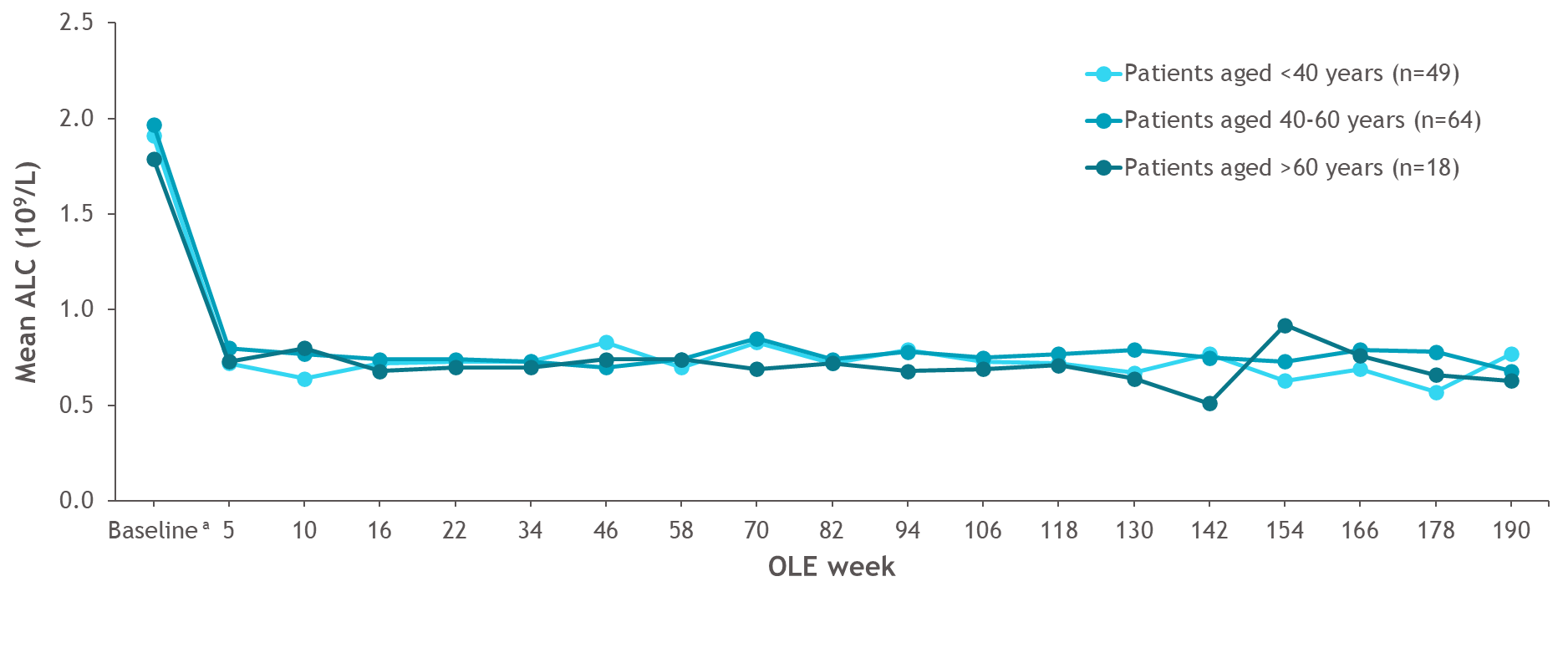
**
